# Adenoid cystic carcinoma of cervix: two cases report and review of the literature

**DOI:** 10.11604/pamj.2015.20.77.5720

**Published:** 2015-01-29

**Authors:** Khadija Benhayoune, Hinde El Fatemi, Abdelaziz Bannani, Abdelilah Melhouf, Toufik Harmouch

**Affiliations:** 1 Laboratory of Surgical Pathology, CHU Hassan II of Fez, Fez, Morocco; 2 Department of Gyneacology I, CHU Hassan II of Fez, Fez, Morocco; 3 Department of Gyneacology II, CHU Hassan II of Fez, Fez, Morocco

**Keywords:** Adenoid cystic carcinoma, cervix, bleeding, cribriform pattern

## Abstract

Adenoid cystic carcinoma of the cervix is a rare and aggressive tumor with fatal outcome. In this paper we report two cases of primary adenoid cystic carcinoma and a review of literature. A 80 years old woman, admitted to our hospital with postmenopausal bleeding and hydrorrhea. Gynealogical examination showed a cervical stenotic with the presence of a tumor processus. Biopsy of cervical growth was done. 80-year-old woman presented with vaginal bleeding with pelvic pain. Physical examination revealed a friable mass in the cervix. Incisional biopsy was performed. In the both cases the diagnosis of adenoid cystic carcinoma of the cercix was confirmed. Adenoid cystic carcinoma of the cervix is clinically and radiologically similar to other tumors of the cervix but the diagnosis can only be made by histological examination.

## Introduction

Adenoid cystic carcinoma (ACC) (also called adenocystic carcinoma, cylindroma or cylindromatous adenocarcinoma) is a malignant epithelial neoplasm derived from the salivary glands and can occur in variety of other sites, such as minor and major glands, lacrymal glands, mucous glands of the aero-digestive tract, skin, breast and lung [1, 2]. In the female reproductive tract, adenoid cystic carcinoma occurs most commonly in the Bartholin gland and rarely in the cervix [1]. It accounts less than 1% of all cervical carcinoma. A very wide spectrum of pathologic entities has been presented as ACC of the cervix. Billroth in 1859 originally defined this lesion as ‘‘tumor composed of small basal cells with slight eosinophilic cytoplasm, hyperchromatic nuclei in central amorphous eosinophilic mass of varying amounts’’. This definition has been extended today to include tumors where spindle or basal cells predominate. Most of the patients are over 60 years of age, and there is a high proportion of black women [3]. It is characterized by slow grouth and high rate of local recurrence. Metastatic spread is usually a long-term complication [4, 5]. Because of the rarety of the disease, no standard treatment has yet been proposed. We report two cases of primary ACC of the cervix and we discuss the clinical, pathological and therapeutic features of disease.

## Patient and observation

### CASE 1

A80 years old postmenopausal moroccan woman (para6, gravida6) admitted to our hospital with postmenopausal bleeding and hydrorrhea. Gynealogical examination showed a cervical stenotic with the presence of a tumor processus whose size is diffuclt to assess. On bimanual recto-vaginal examination we showed that the tumor had invaded both sides of the parametrium. Biopsy of cervical growth was done and sent for histopathological examination.

### CASE 2

**Figure 1:**
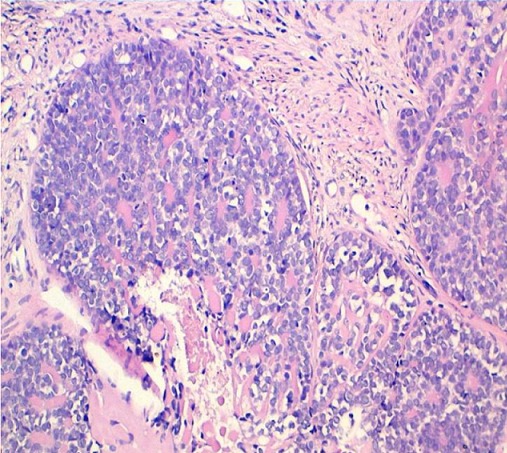
Proliferation composed of small basaloid cells with trabecular structures. Cylindromatous structures are present

**Figure 2:**
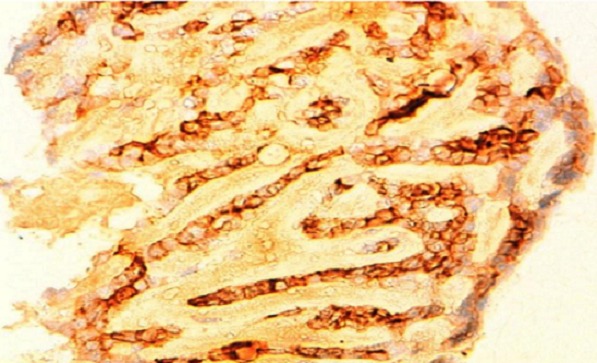
CD117 immunohistochemestry highlighting tumor cells in the adenoid cystic carcinoma

80-year-old woman presented to Department of gynaecology with vaginal bleeding with pelvic pain. Physical examination revealed a friable mass in the cervix. Bimanual pelvic examination showed that vagina, uterus and bilateral adnexal structures are normal. Speculum examination showed an ulcerative and exophytic mass, 4cm in diameter on the anterior lip of the cervix. The lymph nodes are free. Transabdominal pelvic ultrasound reported that the uterus was with homogenous myometrium, indistinct endometrial echo pattern and bilateral adnexal areas were normal. Incisional biopsy was performed. Microscopic examination showed fragmented biopsy with tumor cells disposed in cribriform nests and cords. The tumor cells were small, uniform, composed of dense basophilic nuclei with inconspious nucleoli. Mitotic figures were rarely found (Figure 1). Immunohistochemical study was performed. Tumor cells express receptors ant-CD117, hormone receptors and focally anti-CK5/6, anti-S-100 protein and anti-CD56. A diagnosis of adenoid cystic carcinoma was made (Figure 2).

## Discussion

Adenoid cystic carcinoma pattern is best knowen as neoplasm of the major and minor salivary glands. The first case of adenoid cystic carcinoma of the cervix was reported as cylindroma in 1949 by Paalman and Counseller [2, 6]. There are no studies that have systematically investigated the incidence of ACC. However, it accounts less than 1% of all cervical carcinoma. The most accepted view regarding its origin in the cervix is from ‘‘reserve cells’’ of endocervix [2]. The origin of this disease is still unknown. Although, Human papillomavirus (HPV) infection is belived to be a necessary cause of cervical cancer, its role in the pathogenesis of ACC is not well defined [4, 7]. These tumors usually occurs in postmenopausal women. Though, it has been reported from 24 to 99 years of age, majority of the cases have been reported during 7th to 9th decades of life [2, 8-11]. But, some recent data reported some cases in young woman [4, 12, 13]. Our patient was postmenopausal and multiparous woman. Although, some authors suggested the association between ACC, highparity and black race [4, 14, 15]. Clinical and radiological characteristics of primary ACC of the cervix were similar to those of a squamous cell cancer. The main symptom of disease was the vaginal bleeding [4, 12]. In addition, vaginal discharge and uterine enlargement can be seen. Clinically, it presents as a nonfriable mass on speculum examination, in contrast to the friable growth usually seen in squamous cell carcinoma of cervix. The details of the gross examination of the surgical specimens were not clearly specified. Seemingly, the gross features of these lesions did not differ from those usually seen in ordinary squamous-cell carcinoma of the cervix. Their size varied from small tumors involing portions of the cervix and invading the parametria. The histologic types of the primary carcinoma of the cervix cannot be classified simply as either a squamous or a gland cell lesion (adenocarcinoma). Despite the fact that there were co-existing components of squamous cell metaplasia or carcinoma of the cerix has been found, by means of election microscopic study, to prossess structures of glandular epithelium [16, 17]. This finding suggests that adenoid cystic carcinoma shares the origin from endocervical glands with ordinary cervical adenocarcinoma. The characteristics pathologic features of ACC have been amply documented in several reports. The histologic appearance is varied and includes cribriform, corde-like, glandular and basaloïd patterns, as well as the classical cylindromatous appearance.

Cribriform is the most characteristic pattern in ACC and is composed of polygonal to spindled cells forming numerous duct-like structures that contain extracellular matrices filled with homogenous eosinophilic periodic acid-Schiff (PAS) positive material or granular basophilic material [18, 19]. The pseudocystic spaces stained positively with antibodies to type IV collagen, laminin, heparan sulfate proteoglycan and entractin. In the tubular type, small ductal structures formed by one or two layers of cuboïdal or polygonal tumor cells were intermingled with solid strands of tumor cells. The lamina of these tubules contained an eosinophilic amorphous material. The solid type is characterized by solid sheets with prominent mitotic activity and frequently with central necrosis. Small clusters of slightly larger polygonal cells are occasionally present. This pattern is morphologically least differentiated and is not diagnostic by itself. Perzin et al. [20, 21]described a grading system for ACC based on distinctive histologic patterns: tubular, cribriform and solid. They believed that the three patterns reflect a progression of cellular proliferation and aggressiveness of biologic behavior. The tumor is a assigned to three histologic grades: gradeI,a well-differentiated tumor composed of tubular and cribriform patterns without solid components; grade II: a tumor with a pure cribriform pattern or mixedwith less than 30% of solid aeras and grade III, a tumor with marked predominance of the solid pattern. This tumor has a remarkable tendency for invasion perineural spaces, to the degree that the diagnosis of ACC should be questionned if an adequate sample taken from the periphery of the tumor does not exhibit the feature. This is thought to be caused or promoted by the production of brain- derived neurotopic factor [22]. ACC can also occur in association with other tumor types as an example of squamous or adenocarcinoma tumor components. Immunohistochemically, the tumor cells located in recognizable duct structures express a phenotype similar to that of the the intercalated duct (positive for keratin, S-100 protein and CD117 (C-kit)) and those around pseudocysts have a phenotype suggestive of myoepithelial cell differentiation (positive for S-100 protein and actin and variable for keratin). The patter of c-kit expression in ACC differs with the histologic subtype. Tubular and cribriform variants primarily show C-kit expression in the luminal cell layers. This suggests that myoepithelial cells do not express C-kit as the abluminal cells do not stain [23, 24]. Paradoxically, solid variants show expression in all cells, most of which are considered modified myoepithelial cells [23, 24]. This suggests the role of C-kit overexpression in the aggressive clinical behavior. There is also strong reactivity for basement membrane components, particularly along the inner luminal surface of the pseudocysts [25]. These components include type IV collagen, laminin, their intergin ligands, heparain sulfate proteoglycan (perlecan) and entactin [19, 26, 27]. The basement membrane material also stains for alph-1-antitrypsin [28]. Adenoid cystic carcinomas have also been found to express hormone receptors [29] and about a half of them stain for CD34[30].

The differential diagnosis includes small cell carcinoma, adenoid basal carcinoma and no-keratinizing squamous cell carcinoma. Furthemore, as already stated, ACC is usually strongly immunoreactive for CD117. Important points to remember are that ACC is usually invasive and often associated with perineurial invasion and that mesenchyme-like areas and foci of squamous metaplasia are consistently absent. ACC of the cervix seems to be more aggressive than squamous cell carcinoma of the cervix with higher tendency to local and metastatic recurrence even if diagnosed in their earliest stages [4, 5]. Five years and ten years survival were 37% and 40% respectively [4, 31]. In some series, the recurrence rate was 59% for the tubular tumors, 89% for the classic cribriform lesions and 100% for the solid variety. In another series in which a somewhat similar grading system was used, the cumulative survival rates at 5 years were 39%, 26% and 5%, respectively [32]. The solid or anaplasic type of ACC is associated with a higher incidences in which a conventional ACC undergoes dedifferentiation or high-grade transformation [33], an event that is accompanied by TP53 gene mutation [34]. The solid subtype carries a worse prognosis in terms of the development of distant metastases and overall survival. For the solid subtype, control of local disease is not as important a variable for ultimate survival, due to the rapidity of death from distant metastases. The cribriform subtype demonstrated greater local aggressiveness and a poorer salvage rate as compared with the tubular subtype [20]. Other factor that influences the prognosis of ACC is the anatomic site. The lower survival rate is probably explained by the fact that ACC arising in some difficult region impossible to excise completely [35, 36]. The presence of tumor at the surgical magin appears to be an important factor in predicting prognosis and the size of the tumor appears also to be one of the most important factor in prognosis. Cure is obtained with much frequency in patients with smaller primary lesions [36]. Factors other than histologic grade and location that appear to inflence survival are the invasion of adjacent anatomic structures, deep stromal invasion and the presence of tumor cells within a lymphatic space or capillary [36]. Abnormalities of E-cadhrin expression have been detected, and the claim has been made that reduced expression of this adhesion molecule correlates with an unfavorable prognosis [37]. Because of the rarity of the disease and the absence of prospective studies, no standard treatment has yet been proposed. Most patients were treated as squamous cell carcinoma. Surgery and radiation therapy has been used in the treatment of ACC of the cervix. The best treatment is surgery, principally when the surgical lines of resection could be histologically determined. Radiotherapy proved to be a useful method of treament for surgically treated patients whose surgical borders were indaquate, those who experienced recurrent disease and those considered inoperable [38]. Chemotherapy has very little role in the management of these tumors but has been used for advanced/recurrent/metastatic disease without much success [2, 9, 13].

## Conclusion

Adenoid cystic carcinoma of the cervix is a rare and aggressive tumor with fatal outcome. We must think of this tumor in elderly woman and the presence of perineurial invasion. It has a clinical course influenced principally by the clinical staging and the histologic pattern of growth presented by the tumor. The prognosis is also modified by the size and the presence or absence the tumor at the margins. Surgery is the best treatment of this disease and radiotherapy is an important palliative procedure that prolongs the life of the patients who are not cured by surgery.
